# Differential organ-specific inflammatory response to progranulin in high-fat diet-fed mice

**DOI:** 10.1038/s41598-020-80940-8

**Published:** 2021-01-13

**Authors:** Maki Murakoshi, Tomohito Gohda, Eri Adachi, Saki Ichikawa, Shinji Hagiwara, Yusuke Suzuki

**Affiliations:** Department of Nephrology, Juntendo University Faculty of Medicine, 2-1-1 Hongo, Bunkyo-ku, Tokyo, 113-8421 Japan

**Keywords:** Endocrinology, Nephrology

## Abstract

Progranulin (PGRN) has been reported to bind tumor necrosis factor (TNF) receptor and to inhibit TNFα signaling. We evaluated the effect of augmentation of TNFα signaling by PGRN deficiency on the progression of kidney injury. Eight-week-old PGRN knockout (KO) and wild-type (WT) mice were fed a standard diet or high-fat diet (HFD) for 12 weeks. Albuminuria, markers of tubular damage, and renal mRNA levels of inflammatory cytokines were higher in HFD-fed KO (KO-HFD) mice than in HFD-fed WT (WT-HFD) mice. Body weight, vacuolization in proximal tubules, and systemic and adipose tissue inflammatory markers were lower in the KO-HFD mice than in the WT-HFD mice. The renal megalin expression was lower in the KO mice than in the WT mice regardless of the diet type. The megalin expression was also reduced in mouse proximal tubule epithelial cells stimulated with TNFα and in those with PGRN knockdown by small interfering RNA in vitro. PGRN deficiency was associated with both exacerbated renal inflammation and decreased systemic inflammation, including that in the adipose tissue of mice with HFD-induced obesity. Improved tubular vacuolization in the KO-HFD mice might partially be explained by the decreased expression of megalin in proximal tubules.

## Introduction

Inflammation is an important physiological process that contributes to homeostasis and is a normal bioprotective response to pathogen exposure, cell injury, and stress. However, excessive and prolonged inflammatory response can lead to tissue damage^1^. Therefore, specific regulatory mechanisms limit inflammatory response.

Progranulin (PGRN), a 593-amino acid glycoprotein, is a growth factor involved in embryonic development, tissue repair, tumorigenesis, and inflammation^2^. Studies have demonstrated that PGRN regulates inflammatory responses by counteracting tumor necrosis factor (TNF)-mediated inflammatory signaling pathway^3^ and exerts a renoprotective function in a mouse model of acute renal failure^4^. PGRN has also been shown to contribute to insulin resistance in some metabolic diseases^5^. For example, the administration of PGRN to high-fat diet (HFD)-fed mice led to insulin resistance whereas obesity and insulin resistance induced by HFD were suppressed in PGRN-deficient mice^5^. Several studies have reported increased circulating PGRN concentrations in patients with metabolic diseases such as diabetes and obesity^6,7^. Additionally, we have previously shown that circulating PGRN concentrations are significantly increased in patients with type 2 diabetes and renal functional decline^8^. These findings raise the possibility that PGRN may exert beneficial or harmful effects depending on the affected organ or pathological condition.

TNFα is a well-known inflammatory cytokine involved in the progression of kidney disease^9^ and binds two TNF receptors (TNFRs), TNFR1 and TNFR2, to mediate downstream signaling. TNFRs are solubilized by the proteolytic cleavage of its extracellular domain^10^. We and others have previously reported that soluble TNFRs are strong biomarkers associated with renal functional decline in patients with a variety of renal diseases, such as diabetic kidney disease^8,11–16^. Further, PGRN has been shown to directly bind TNFR with a binding affinity equal to or higher than that of TNFα, which leads to the inhibition of the TNF/TNFR signaling pathway^17,18^. Several studies have reported that TNFα is associated with the development/progression of kidney diseases through the activation of TNF/TNFR pathway^19^. However, studies investigating the role of PGRN in inflammation in renal disease are limited^7,20^. This study aimed to determine whether PGRN plays a beneficial or detrimental role in two different organs, kidneys and the adipose tissue, using PGRN-deficient mice with HFD-induced chronic inflammation.

## Results

### Phenotypic characterization of experimental groups

The phenotypic characteristics of 20-week-old PGRN-knockout (KO) and wild-type (WT) mice fed an HFD or normal SD for 12 weeks are shown in Table 1. There were no significant differences in body weight, food intake, albumin/creatinine ratio (ACR), and the levels of urinary kidney injury molecule-1 (KIM-1), serum TNFα (sTNFα), serum TNFR1 (sTNFR1), serum TNFR2 (sTNFR2), and urinary 8-Oxo-2′-deoxyguanosine (8-OHdG) between the SD-fed PGRN-KO (KO-SD) group and the SD-fed WT (WT-SD) group. In contrast, all these parameters were significantly higher in the HFD-fed WT (WT-HFD) group than in the WT-SD group.

**Table 1:** General and biochemical parameters of experimental groups after 12-weeks of HFD.

Mouse	WT		PGRN-KO		Group contrast	
Type of food	SD	HFD	SD	HFD	KO (p^a^)	HFD (p^b^)
Body weight (g)	28.5 ± 1.4	49.6 ± 5.5	30.0 ± 1.7	40.6 ± 3.6	0.03	< 0.01
Food intake (kcal/day)	10.7 ± 0.6	14.1 ± 1.1	11.9 ± 0.7	13.8 ± 1.3	0.30	< 0.01
ACR (mg/gCr)	8 (7, 9)	38 (33, 40)	9 (9, 12)	67 (56, 72)	< 0.01	< 0.01
KIM-1 (pg/mgCr)	5310 (4866, 5437)	9456 (8179, 15,144)	5615 (4041, 7230)	19,004 (17,466, 29,859)	0.04	< 0.01
sTNFα (pg/mL)	0.74 (0.64, 1.32)	3.86 (1.93, 4.25)	1.03 (0.89, 1.48)	2.43 (1.59, 2.82)	0.78	< 0.01
sTNFR1 (pg/mL)	1074 (1046, 1153)	1764 (1476, 1851)	1043 (1014, 1086)	1259 (1211, 1282)	0.06	< 0.01
sTNFR2 (pg/mL)	5486 (5071, 5901)	10,662 (7658, 11,673)	6012 (5850, 6447)	8305 (7196, 8687)	0.45	< 0.01
8-OHdG (mg/gCr)	82 (78, 87)	172 (146, 198)	77 (69, 85)	81 (74, 105)	0.01	< 0.01

Two-way ANOVA was used to evaluate the individual effects of the presence/absence of PGRN and diet type. Body weight, food intake, and ACR: n = 6–7/group; KIM-1, sTNFα, and sTNFR2: n = 8–11/group; sTNFR1 and 8-OHdG: n = 4/group.

Data are expressed as means ± SD or median (quartiles).

*ACR* albumin/creatinine ratio, *sTNFα* serum TNFα, *sTNFR1* serum TNFR1, *sTNFR2* serum TNFR2, *WT* wild-type C57BL/6 J, *KO* PGRN homozygous knockout, *HFD* high-fat diet.

^a^By ANOVA to determine the effect of PGRN status.

^b^By ANOVA to determine the effect of diet type.

**Figure 1:**
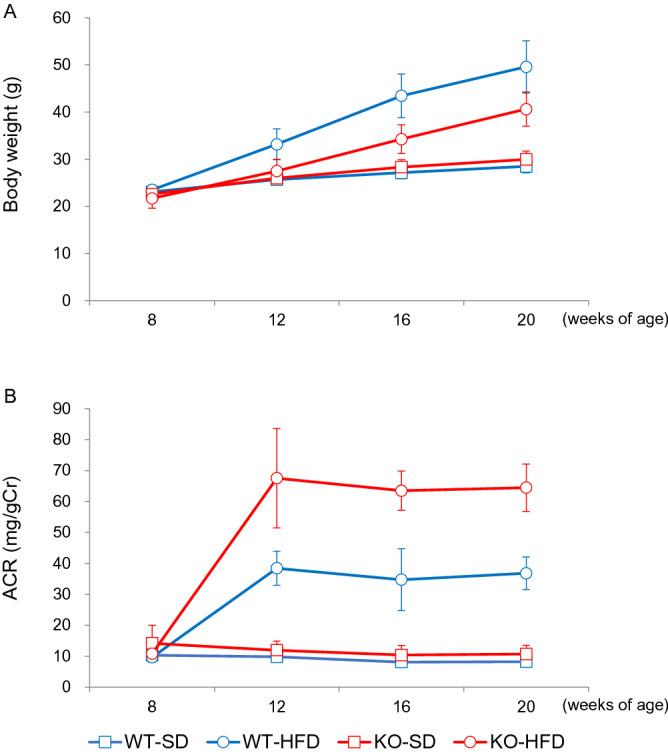
Mean body weight and urinary ACR in each experimental group. (**A**) Body weight was measured at 8, 12, 16, and 20 weeks of age. The KO-HFD group was protected against obesity despite the lack of difference in food intake between the KO-HFD and WT-HFD groups. (**B**) Urinary ACR, measured at 12, 16, and 20 weeks of age, is highest in the KO-HFD group at all timepoints. Data are expressed as means ± standard deviation.

Despite the similar food intake (Figure S1), the body weights at 12, 16, and 20 weeks of age were higher in the WT-HFD group than in the HFD-fed KO (KO-HFD) group (Fig. 1A). Two-way ANOVA revealed significant main effects of PGRN status and diet type on body weight at 20 weeks of age (Table 1). The combined main effects of PGRN status and diet type resulted in the highest body weight in the WT-HFD group (interaction in two-way ANOVA, *p* < 0.01). The glomerular injury marker ACR, which was determined at 12, 16, and 20 weeks of age (Fig. 1B), and the tubulointerstitial injury marker urinary KIM-1, which was measured at 20 weeks of age, were highest in the KO-HFD group. However, the levels of sTNFα, sTNFR1, sTNFR2, and urinary 8-OHdG were highest in the WT-HFD group.

All measured parameters included in Table 1 were significantly different between the SD and HFD groups. Conversely, body weight, ACR, urinary KIM-1, and urinary 8-OHdG were significantly different between the WT and KO groups. The expected association of HFD with higher sTNF, sTNFR1, and sTNFR2 levels was present in the current study. However, none of these parameters were associated with the absence of PGRN.

### PGRN expression in proximal tubules and adipose tissue

**Figure 2:**
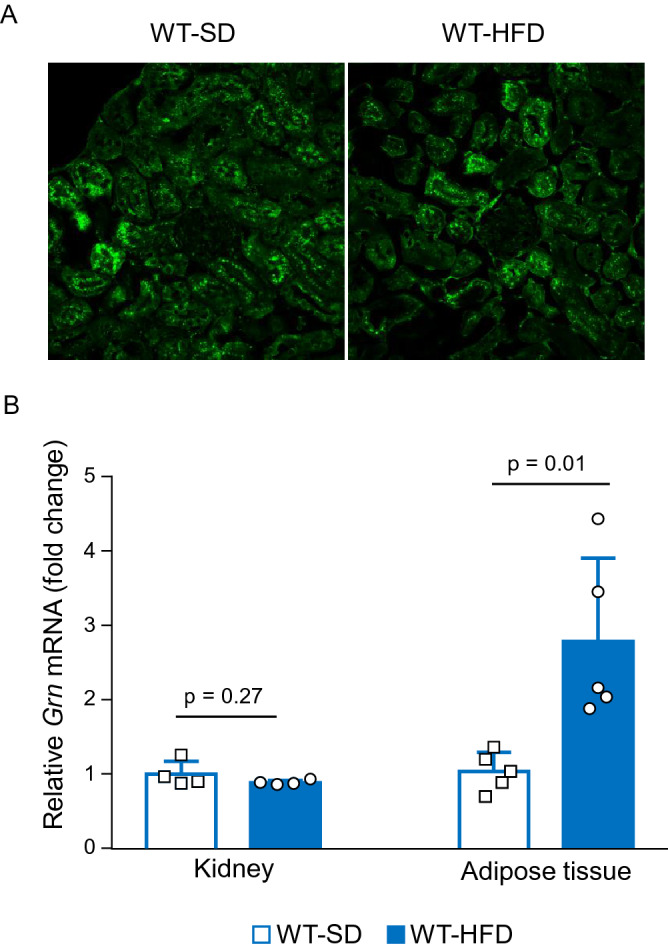
*Grn* mRNA and protein expression levels in the kidney and adipose tissue of the WT-SD and WT-HFD groups. (**A**) Immunofluorescence staining for PGRN shows PGRN expression primarily localized to the tubules. (**B**) *Grn* mRNA expression levels in the kidney and adipose tissue by real-time PCR. There is no difference in the renal *Grn* mRNA expression level between the WT-SD and WT-HFD groups. Conversely, the adipose tissue *Grn* mRNA expression level is significantly higher in the WT-HFD group than in the WT-SD group. Student’s unpaired *t* test.

Immunofluorescence staining showed that PGRN was mainly localized in the proximal tubules (Fig. 2A). There was no significant difference in renal *Grn* (PGRN) mRNA expression levels at 20 weeks of age between the WT-HFD and WT-SD groups. However, the *Grn* mRNA expression level was significantly higher in the adipose tissue of the WT-HFD group than that of the WT-SD group (Fig. 2B), in agreement with a previous study^5^. Immunofluorescence staining confirmed the absence of PGRN expression in PGRN-KO mice (Figure S2).

### The absence of PGRN ameliorates vacuolar formation in proximal tubules of HFD-fed mice

**Figure 3:**
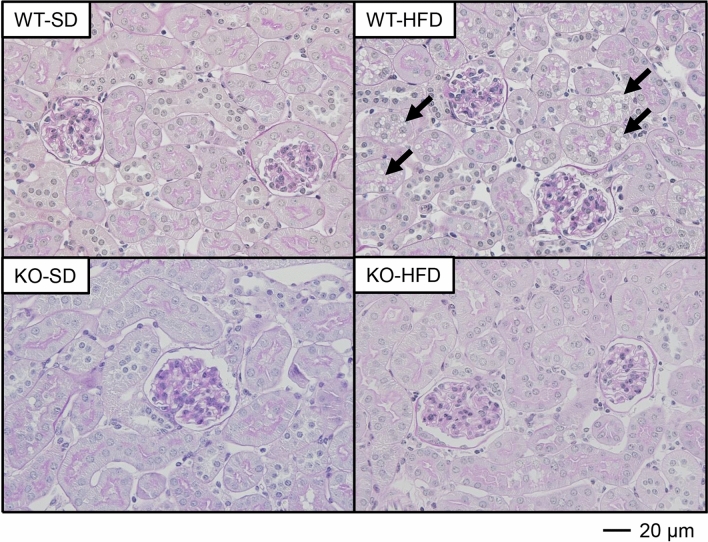
PAS staining of kidney tissue. Vacuolization is observed in the proximal tubules of the WT-HFD group (closed arrowheads) but not of the KO-HFD group by PAS staining of kidney sections from 20-week-old mice (400 ×, scale bar: 20 µm).

**Figure 4:**
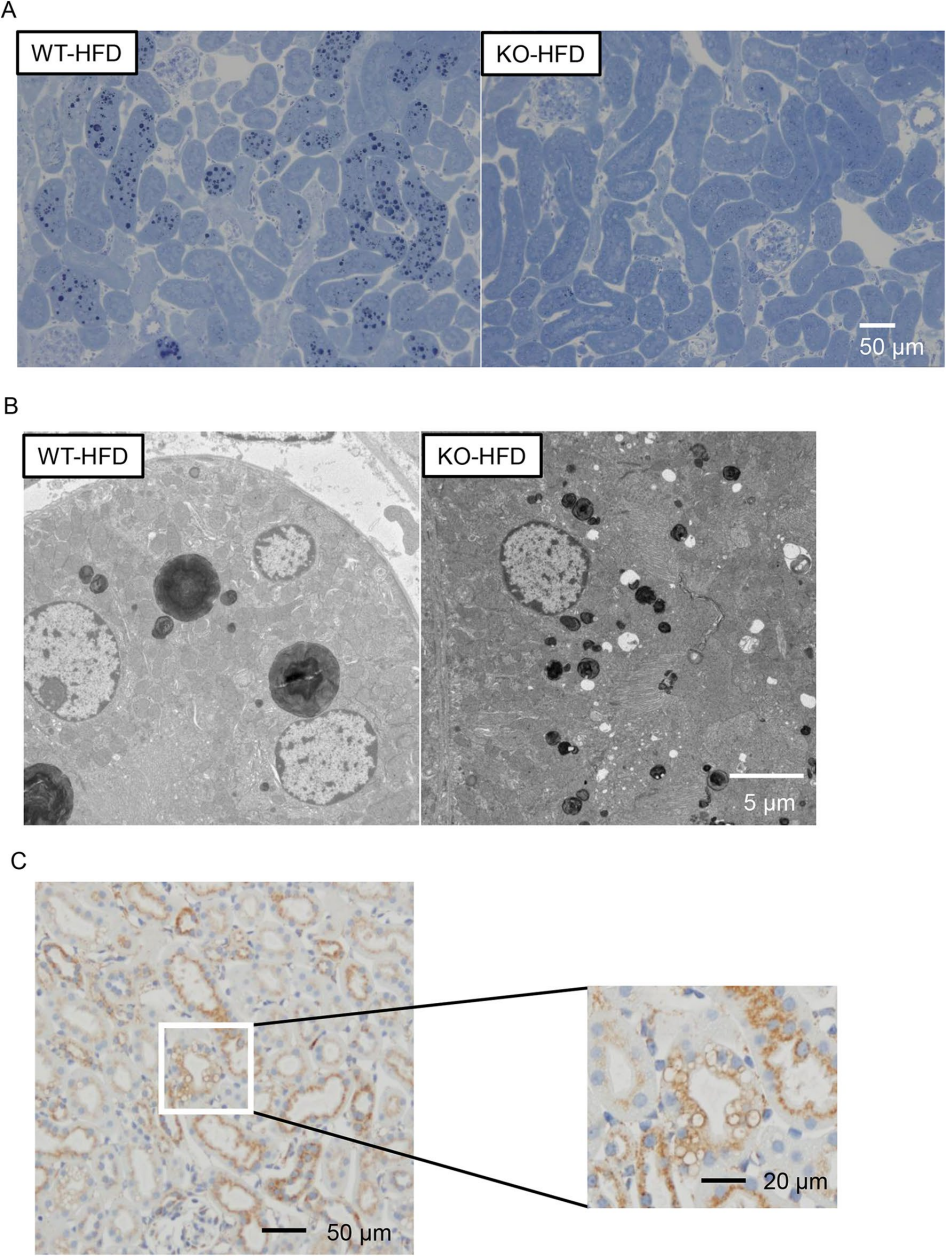
Vacuolation in the proximal tubules of WT-HFD and KO-HFD mice. (**A**) Toluidine blue staining (200 ×, scale bar: 50 µm) and (**B**) electron microscopy of the proximal tubules (1200 ×, scale bar: 5 µm). Aggregation with strong toluidine blue staining is observed in some proximal tubules in the WT-HFD group, whereas the aggregation is rarely observed in the KO-HFD group. Electron-dense multilamellar whirl structures are present in the cytoplasm of tubules in both groups. The accumulation is more extensive in the WT-HFD group than in the KO-HFD group. (**C**) Immunohistochemical staining for LAMP1 in the proximal tubules of the WT-HFD group shows that LAMP1 surrounds vacuolar membranes (200 × , scale bar: 50 µm).

The histopathological examination of kidney tissue samples to assess morphological changes in each group revealed that the cytosolic vacuolar formation in proximal tubules was the most distinct histologic feature of the WT-HFD group (Fig. 3). Of note, almost no vacuolation was observed in the proximal tubules of mice in the KO-HFD group. The vacuoles did not stain with Oil Red O (Figure S3), suggesting that these vacuolar structures did not contain neutral triglycerides. Indeed, the vacuolar contents were strongly stained with toluidine blue in the kidney tissue samples fixed with glutaraldehyde and osmium tetroxide (OsO_4_) (Fig. 4A). Electron microscopy showed that the vacuoles contained OsO4-stained multilamellar whirl structures (Fig. 4B). These vacuoles were surrounded by lysosomal-associated membrane protein 1 (LAMP1) (Fig. 4C), a lysosome marker, indicating that the vacuoles could be intralysosomal phospholipid accumulation.

### Differential inflammatory response to PGRN absence between kidney and adipose tissue

**Figure 5:**
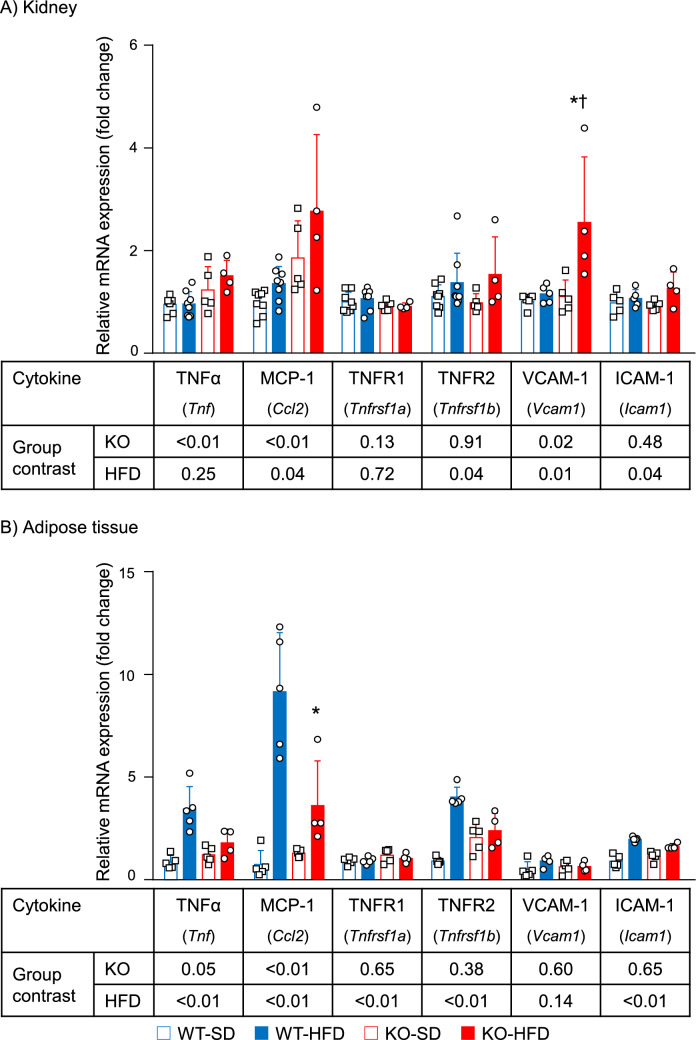
Kidney and adipose tissue mRNA expression levels of inflammatory cytokines in each group. (**A**) Kidney. (**B**) Adipose tissue. Two-way ANOVA was used to evaluate the individual effects of the presence/absence of PGRN and diet type and their interactions. In the kidney, *Ccl2* and *Vcam1* mRNA expression levels are significantly associated with both PGRN status and diet type. In contrast to the effect on kidney mRNA expression levels, PGRN status and diet type enhance adipose tissue *Ccl2* mRNA expression levels in the WT-HFD group. For the mRNA expression levels of *Vcam1* in the kidney and *Ccl2* in the adipose tissue, main effects were confirmed for PGRN status and diet type and for an interaction between PGRN status and diet type based on post hoc Tukey’s honestly significant difference test. Data are shown as fold changes compared to the WT-SD group, *p < 0.05 versus WT-HFD, ^†^*p* < 0.05 versus KO-SD.

HFD has been extensively shown to promote low-grade chronic inflammation. Therefore, we investigated whether PGRN deficiency exacerbated renal inflammation by measuring the mRNA expression levels of *Tnf* (TNFα), *Tnfrsf1a* (TNFR1), *Tnfrsf1b* (TNFR2), *Ccl2* (MCP-1), vascular cell adhesion molecule 1 (*Vcam1;* VCAM-1), and intercellular adhesion molecule 1 (*Icam1;* ICAM-1) by real-time PCR (Fig. 5A). In the kidney, *Ccl2* and *Vcam1* mRNA expression levels were significantly associated with both PGRN status and diet type. The combined main effects of PGRN status and diet type resulted in the highest *Vcam1* mRNA expression levels in the KO-HFD group compared with the WT-HFD group (interaction in two-way ANOVA, *p* = 0.04). *Tnf* mRNA expression level was significantly associated only with PGRN status, and *Tnfrsf1b* and *Icam1* mRNA expression levels were significantly associated only with the diet type. There were no significant main effects by PGRN status and diet type on *Tnfrsf1a* mRNA expression level. Conversely, the mRNA expression levels of all inflammatory markers except *Vcam1* were significantly different in the adipose tissue between the SD and HFD groups. *Ccl2* mRNA expression level was significantly associated with both PGRN status and diet type. The combined main effects of PGRN status and diet type resulted in the highest adipose tissue mRNA levels of *Tnf*, *Ccl2*, and *Tnfrsf1b* in WT-HFD group compared with the KO-HFD group (all interaction effects by two-way ANOVA, *p* < 0.01) (Fig. 5B).

### Low megalin expression in the proximal tubules of PGRN-KO mice

**Figure 6:**
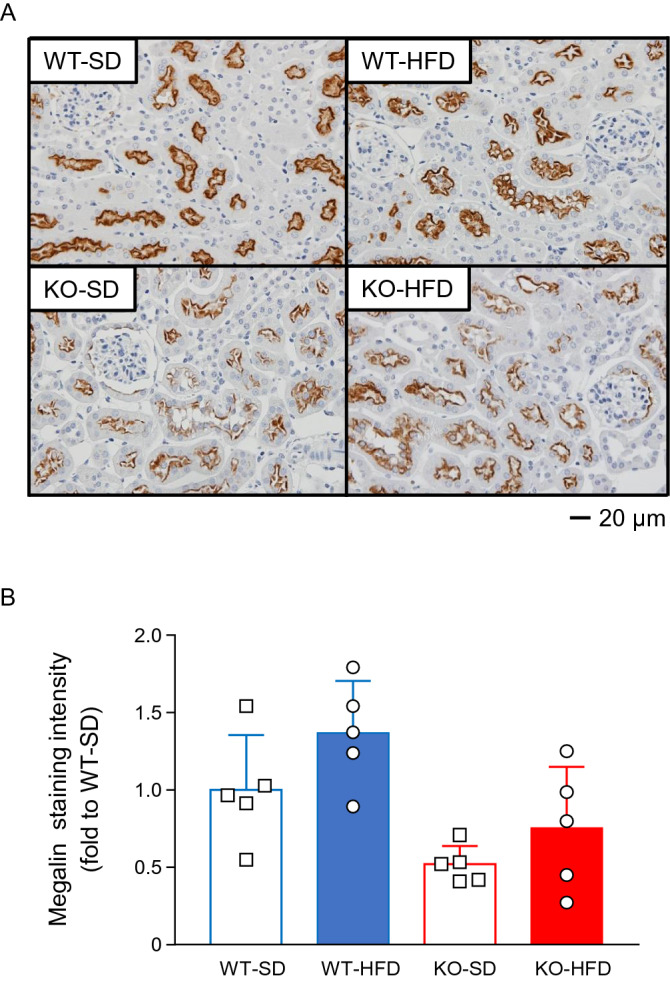
Protein expression levels of megalin in each group. (**A**) Immunohistochemistry for megalin (400 × , scale bar: 20 µm). Staining is primarily localized to the proximal tubules. (**B**) Staining intensity of megalin protein in the mouse kidney is presented as fold changes compared to the WT-SD group. Two-way ANOVA shows a significant main effect of only PGRN status on megalin protein expression. *p* < 0.01 for PGRN status; *p* = 0.64 for diet type.

Reabsorption of urinary proteins in proximal tubules is primarily mediated by megalin^21^. Given that albuminuria was increased in the PGRN-KO mice, we next evaluated megalin protein expression and found that there was a significant main effect of PGRN status alone on reduction in megalin level (Fig. 6).

### PGRN absence reduces megalin expression through an inflammatory response

**Figure 7:**
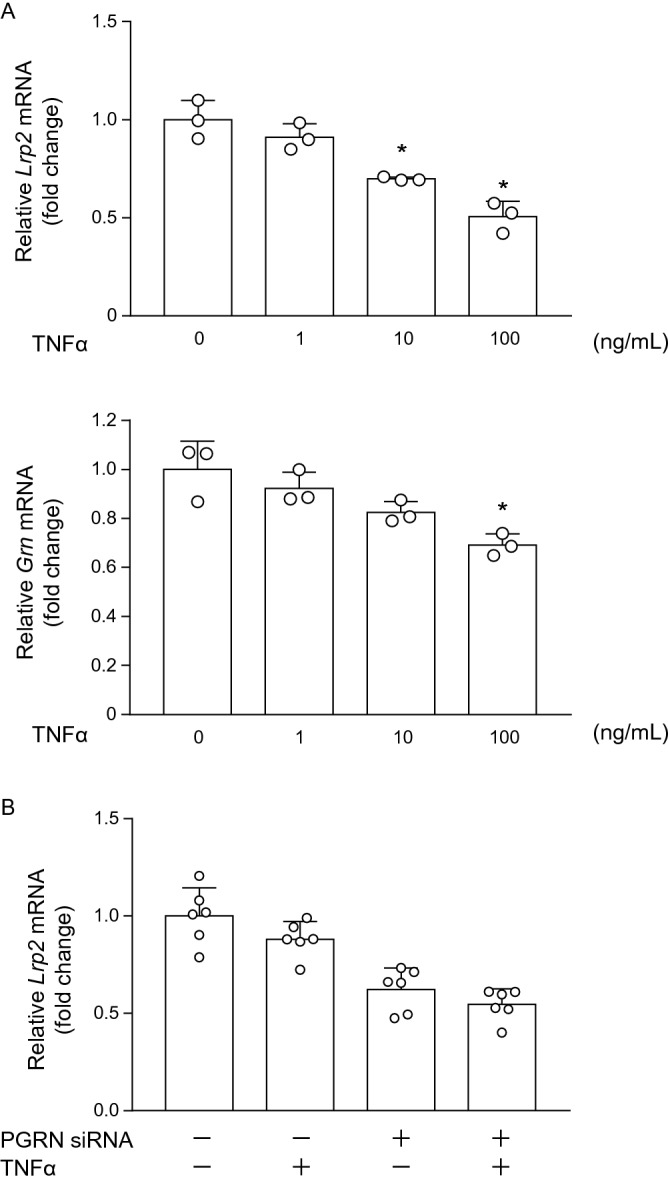
mRNA expression levels of *Lrp2* and *Grn* in cultured mouse PTECs stimulated with TNFα. (**A**) The mRNA expression levels of *Lrp2* and *Grn* are reduced in a dose-dependent manner in PTECs stimulated with TNFα. Tukey’s honestly significant difference test. *p < 0.05 versus no TNFα stimulation. (**B**) Effects of PGRN siRNA on megalin expression in PTECs stimulated with 10 ng/mL TNFα. Two-way ANOVA shows significant main effects of PGRN knockdown and TNFα stimulation on the expression of megalin (*p* < 0.01 and *p* = 0.04, respectively), without significant interaction (*p* = 0.64), showing that knocking down PGRN reduces megalin expression.

Based on our findings showing the increased renal mRNA expression levels of inflammatory cytokines and the decreased renal protein levels of megalin in PGRN-KO mice, we next investigated the relationship between PGRN and megalin in proximal tubules in the presence of inflammation. To that end, PTECs were stimulated with TNFα, which led to increased mRNA expression levels of TNFα-related inflammatory molecules, such as *Ccl2* and *Tnfrsf1b*, in PTECs (Figure S4). When the cultured PTECs were stimulated with TNFα, the mRNA expression levels of *Lrp2* (megalin) and *Grn* decreased in a concentration-dependent manner (Fig. 7A).

Next, PTECs transfected with small interfering RNAs (siRNAs) targeting *Grn* were stimulated with 10 ng/mL TNFα. The transfection by PGRN siRNAs reduced the *Grn* mRNA expression levels by approximately 70% compared to the PTECs transfected with negative control siRNA (Figure S5). Two-way ANOVA revealed significant main effects of PGRN knockdown and TNFα stimulation on megalin expression but there was no significant interaction between PGRN knockdown and TNFα stimulation (Fig. 7B). Collectively, these data suggested that these two factors acted independently and that renal inflammation exacerbated by PGRN absence in vivo reduced megalin expression in proximal tubules.

## Discussion

In the present study, we demonstrated that PGRN deficiency was associated with exacerbated local renal inflammation in mice with HFD-induced obesity. However, the cytosolic vacuolar formation in proximal tubules was dramatically improved in the HFD-fed PGRN-KO mice compared to the HFD-fed WT mice. Conversely, systemic inflammation, including that in the adipose tissue, was exacerbated in the HFD-fed WT mice compared with the HFD-fed PGRN-KO mice.

PGRN, which has anti-inflammatory properties, is widely expressed throughout the body and is considered to play a role in wound healing and inflammation. Specifically in kidney diseases, induction of acute renal injury by ischemia–reperfusion has been shown to result in augmented kidney damage in PGRN-KO mice compared with the WT mice^4^. In the present study, we evaluated the role of PGRN in chronic rather than acute renal injury using a chronic renal inflammation model of HFD-induced obesity. The lack of statistical difference in the renal mRNA expression levels of inflammatory cytokines between the WT-SD and WT-HFD groups indicated that HFD-induced obesity was not as severe in this experimental model of renal inflammation. Nevertheless, the mRNA expression levels of inflammatory cytokines were higher in the KO-HFD mice than in the WT-HFD mice, indicating that PGRN deficiency was involved in exacerbated renal inflammation in mice with HFD-induced obesity.

PGRN has been proposed to directly bind TNFRs and alter the TNFα/TNFR interaction^17^. Tang et al. have demonstrated that PGRN binds TNFR2 with a higher binding affinity and blocks TNFα signaling in human chondrocytes using a co-immunoprecipitation assay^17^. We therefore measured serum and renal mRNA expression levels of TNFα, TNFR1, and TNFR2 in PGRN-KO mice. Two-way ANOVA revealed a significant main effect of PGRN status on *Tnf* mRNA expression level in the absence of an interaction between PGRN status and diet type indicating that PGRN status resulted in increased *Tnf* mRNA expression. Conversely, among all groups, WT-HFD mice had the strongest systemic inflammation, based on the levels of sTNFα, sTNFR1, and sTNFR2, leading to the question of which serum inflammation markers reflect systemic inflammation. Matsubara et al. reported that HDF-fed PGRN-KO mice exhibited improved HFD-induced insulin resistance which was observed in WT mice^5^. Several clinical studies have also shown that serum PGRN is associated with obesity, insulin resistance, decreased renal function, and inflammatory factors^6,8,22,23^. In the present study, HFD-induced obesity in WT mice led to increased PGRN expression in the adipose tissues but was not associated with any change in the kidney. The mRNA expression levels of inflammatory cytokines were higher in the adipose tissue of WT-HFD mice than that of the KO-HFD mice. Furthermore, the extent of obesity was milder in the KO-HFD mice than in the WT-HFD mice. Baseline PGRN protein expression has been reported to be lower in the kidney than in other organs such as the liver^24^. Therefore, circulating TNFα and TNFR2 levels might reflect systemic inflammation which might affect body weight and insulin resistance. We also showed that urinary 8-OHdG level was lower in the KO-HFD group than in the WT-HFD group. Li et al.^25^ reported that urinary 8-OHdG levels were significantly higher in the diabetic db/db mice than in the control db/m + mice whereas the 8-OHdG levels in the liver and kidney nuclear DNA were not significantly different between the db/db and db/m + mice. Therefore, urinary 8-OHdG might reflect systemic oxidative stress related to systemic inflammation.

Inflammation due to PGRN absence might be different between the kidney and adipose tissue. Studies have revealed abnormal accumulation of lipofuscin granules, accompanied by significantly increased p62, in specific brain regions of mice with PGRN deficiency^26,27^, suggesting the impairment of autophagy-lysosomal system. Autophagy is closely linked to inflammation, in part through its regulation of adipokine production. It is clear that protein levels of autophagy-associated genes are increased in adipose tissues of humans with obesity^28^; however, impaired autophagy with decreased expression of autophagy genes in the liver of obese mice has been shown to be linked to insulin resistance^29^, suggesting that the expression of autophagy genes might differ across tissues. Yoo et al.^30^ reported that PGRN administration improved inflammation and fibrosis and reduced steatosis and hepatocellular injury, whereas Liu et al.^31^ showed that PGRN administration attenuated hepatic insulin sensitivity and hepatic autophagy. These findings illustrate that the action of PGRN might vary depending on the pathology even within the same organ, which warrants further investigations.

In the present obesity model with chronic low-grade inflammation, the absence of PGRN exacerbated kidney injury, although the tubular vacuolation observed in HFD-fed WT mice was improved. Kuwahara et al. reported the presence of vacuoles exclusively in megalin-expressing proximal tubules, which was rarely observed in HFD-fed mosaic megalin-KO mice with approximately 60% KO of megalin expression^32^. Megalin, a 600-kDa glycosylated receptor belonging to the low-density lipoprotein receptor family, is highly expressed in the apical membranes of proximal tubules. Megalin plays a critical role in the reabsorption, i.e., endocytosis, of glomerular-filtered proteins including albumin^33^, the main binding protein for fatty acids in extracellular fluids. Since fatty acids mediate cellular lipotoxicity^34^, megalin might also play a role in mediating renal lipotoxicity. Megalin might be involved in renal tubular vacuolation, considering the relatively low megalin expression in the kidney of PGRN-KO mice compared to the WT mice.

The mechanism of reduced megalin expression in PGRN-KO mice is not clear. Circulating lipopolysaccharide levels are usually increased in patients with type 2 diabetes and/or obesity, and lipopolysaccharide has been reported to suppress megalin expression through the induction of TNFα expression in cultured PTECs^35^. In the kidney of PGRN-KO mice, inflammation markers, including TNFα, tended to be increased whereas TNFα stimulation reduced megalin expression in PTECs, which was further decreased by RNA interference of PGRN. Although we did not investigate the mechanism underlying PGRN-associated reduction in megalin, PGRN was reported to bind sortilin to form an endocytosis-mediated complex that mediates the delivery of PGRN to the endosomal/lysosomal pathway in neurons^36^. Similar to megalin, sortilin is a multiligand receptor that transports proteins from the Golgi apparatus to endosomes, secretory vesicles, and cell membrane. These two multiligand receptors might have similar functions in proximal tubules. For example, several studies on Fabry disease, a typical lysosomal storage disease (LSD), reported that the uptake of α-galactosidase A in podocytes was mediated by M6PR, megalin, and sortilin whereas its uptake in proximal tubules was primarily mediated by megalin^37,38^. Similar to sortilin in neurons, megalin might mediate the delivery of PGRN to the endosomal/lysosomal pathway in the kidney. The results of the present study suggest that the reduced megalin expression triggered by PGRN deficiency ameliorates tubule vacuolation due to endosomal/lysosomal dysfunction caused by megalin-mediated endocytosis in proximal tubules.

The discovery that patients with homozygous *Grn* mutations develop LSD has drawn attention to the localization and role of PGRN in the lysosome^39^. PGRN functions as an intracellular chaperone, and its deficiency leads to lipid accumulation in various LSDs. However, our analyses showing reduced phospholipid accumulation in the kidney tissue of HFD-fed PGRN-KO mice compared to the HFD-fed WT mice contradict previous reports showing that PGRN deficiency enhanced lipid accumulation in LSDs^40,41^. In the present study, PGRN deficiency reduced the size of abnormally large deposits accumulating in the proximal tubules of HFD-fed PGRN-KO mice. One potential explanation for this finding is the reduced megalin expression due to the reduced uptake of albumin-bound fat into the proximal tubules.

The present study has several limitations. First, we examined the effect of PGRN in mice with constitutive PGRN KO and histological changes might be affected by changes in systemic metabolism associated with PGRN deficiency. In addition, the coexistence of exacerbated renal inflammation and improvement in renal vacuolation appear to contradict general conventions on renal disorders. Our model has not been validated beyond the 12-week experimental period; therefore, it remains unclear whether renal failure might develop earlier in mice with augmented inflammation or in those with vacuolation. Longer-term experiments and systemic evaluation might be necessary to further validate our findings. Second, we measured only the mRNA expression levels of inflammation markers in the kidney and adipose tissue. There is generally a poor correlation between the mRNA and corresponding protein levels. Although differentially expressed mRNAs might be more closely correlated with protein products than non-differentially expressed mRNAs^42^, the degree of correlation between mRNA and protein expression levels may vary among different cytokines/chemokines^43^. Moreover, we did not assess changes in the levels of anti-inflammatory molecules, which should be elucidated in future studies.

In conclusion, PGRN deficiency was associated with worse renal inflammation in the presence of improved systemic inflammation, including that in adipose tissue, in mice with HFD-induced obesity. Improved tubular vacuolization in HFD-fed PGRN KO mice might be partially explained by the decreased expression of megalin in proximal tubules.

## Methods

### Animals

PGRN heterozygous KO mice, developed by Kayasuga et al.^24^, were purchased from RIKEN BioResource Center (Ibaraki, Japan). Genotyping of PGRN homozygous KO mice was performed as described previously^24^. WT C57BL/6J mice were used as controls. Mice were individually housed in plastic cages with free access to food and water throughout the experiment. Specifically, 8-week-old mice were randomly divided into four groups to be fed the HFD (WT-HFD and KO-HFD groups; 5.2 kcal/g, 60% of calories from fat [35%/g]; D12492; Research Diets, New Brunswick, NJ, USA) or the standard diet (SD) (WT-SD and KO-SD groups; 3.39 kcal/g, 4.6/g; CE-2; CLEA Japan, Tokyo, Japan) for 12 weeks. All mice were maintained in the same room under specific pathogen-free conditions with a regular 12-h light/dark cycle and a temperature controlled at 24 ± 1 °C. At 20 weeks of age, all mice were sacrificed under intraperitoneally anesthesia with 50 mg/kg sodium pentobarbital to obtain kidney, epididymal white adipose tissue, and blood samples. Blood samples were collected from the left ventricle. All animal experiments were approved by the Ethics Review Committee for Animal Experimentation of Juntendo University Faculty of Medicine (document no. 1312), and all animals were treated according to the guidelines for animal experimentation of Juntendo University in Tokyo, Japan.

### Biochemical analyses

ACR, body weight, and food intake were measured at 8, 12, 16, and 20 weeks of age. As described previously^44^, 24-h urine samples were collected using a mouse metabolic cage (CLEA Japan). Urinary albumin and creatinine were measured by immunoassays using a DCA 2000 Vantage analyzer (Siemens Healthcare, Erlangen, Germany). Enzyme-linked immunosorbent assays were used to measure sTNFα, sTNFR1, sTNFR2, urine KIM-1 (R&D Systems, Minneapolis, MN, USA), and urine 8-OHdG (JaICA, Shizuoka, Japan).

### Polymerase chain reaction (PCR)

Real-time PCR was used for gene expression analysis and performed as previously described^44^. The RNeasy Mini kit (Qiagen, Hilden, Germany) was used for total RNA purification according to the manufacturer’s instructions. TaqMan real-time PCR was performed to evaluate relative mRNA expression (Applied Biosystems, Foster City, CA, USA). Expression levels of *Grn, Lrp2*, *Tnf*, *Tnfrsf1a*, *Tnfrsf1b*, *Ccl2*, *Vcam1*, and *Icam1* in kidney and adipose tissue were measured using commercially available assays from Applied Biosystems. All data were presented as relative mRNA expression levels using the 2^−ΔΔ*C*T^ method^45^. For each gene, average of the threshold cycle (*C*_T_) was subtracted from the corresponding average *C*_T_ for glyceraldehyde 3-phosphate dehydrogenase (*Gapdh*) for each sample to obtain Δ*C*_T_. *Gapdh* was considered as a suitable reference gene for both kidney and white adipose tissue in rodent obesity models^46,47^. Fold increases in other groups compared with the WT-SD group were calculated using the 2^−ΔΔ*C*T^ method.

### Light microscopy and immunohistochemical staining

As described previously^48^, the animals were perfused with 4% paraformaldehyde through the left ventricle and kidneys were collected. Sagittal kidney sections were cut to 4 μm, embedded in paraffin, and stained with periodic acid-Schiff reagent. Immunohistochemical analyses were performed using the following commercially available antibodies: polyclonal sheep anti-PGRN antibody (R&D Systems), monoclonal mouse anti-megalin antibody (Santa Cruz Biotechnology, CA, USA), and polyclonal rat anti-LAMP1 antibody (Abcam, Cambridge, UK). KS400, a computer-aided manipulator (Carl Zeiss Vision, Munich, Germany), was used to quantitatively measure the tubular area. More than 20 consecutive sections of each mouse kidney were randomly selected by scanning from the outer cortex and were examined.

### Electron microscopy

Tissues were fixed with 2% glutaraldehyde in 0.1 mol/L phosphate buffer and post-fixed in 1% OsO_4_, as described previously^44^. The tissues were then cut using a regular diamond knife into semi- and ultrathin sections. Semithin sections of 1-μm thickness stained with 1% toluidine blue were used for light microscopy. Ultrathin sections were collected on 100-mesh copper grids and double stained with 4% uranyl acetate and lead citrate.

### Cell culture

The mProx 24 cells, a murine proximal tubule epithelial cells (PTEC) line, were cultured as described previously^20,49,50^. Cells were cultured in K-1 medium supplemented with 10% fetal bovine serum. All experiments were repeated at least three times for each condition. In all experiments, cells between passages 11 and 25 were used.

The cells were incubated with murine TNFα (1–100 ng/mL) (Merck, Darmstadt, Germany) for 24 h to confirm the direct effect of TNFα on the regulation of PGRN and megalin expression in PTECs.

### siRNA transfection

Cultured PTECs (5 × 10^5^) were seeded in six-well plates and incubated for 24 h. *Grn* was silenced by incubating with a Dharmacon SMARTpool reagent (Thermo Scientific, Pittsburgh, PA), which comprised four *Grn*-specific siRNAs in a single reagent pool to reduce potential off-target issues, and cells incubated with a nontargeting siRNA comprised the negative control group, as previously described^49,51^. The transfection complex included a final concentration of 75 nmol/L of each siRNA and DharmaFECT 4 transfection reagent (Thermo Scientific). Transfection medium containing FCS was removed from the culture after 24 h of incubation. Transfected cells were incubated with K-1 medium supplemented with 1% fetal bovine serum for 12 h, followed by treatment with 10 ng/mL TNFα for an additional 24 h.

### Statistical analysis

Continuous variables with a normal distribution were expressed as means ± standard deviation. Variables with a skewed distribution were handled as continuous variables after common logarithmic transformation and presented as medians (25%–75% interquartile range). All data were tested for equality of variances by the Shapiro–Wilk test. Comparisons between two parameters were analyzed by Student’s unpaired *t* test. Data with two independent variables were analyzed by two-way analysis of variance (ANOVA). In cases with a statistically significant interaction between two independent variables on the dependent variable, post hoc Tukey’s honestly significant difference test was performed. *p* values less than 0.05 were defined to indicate statistical significance. All statistical analyses were performed using the SPSS software (version 23; SPSS, Chicago, IL, USA).

## Supplementary information

Supplementary Figures.

Supplementary Legends.

## References

[1] Chen L (2018). Inflammatory responses and inflammation-associated diseases in organs. Oncotarget.

[2] Jian J, Li G, Hettinghouse A, Liu C (2018). Progranulin: a key player in autoimmune diseases. Cytokine.

[3] Liu CJ, Bosch X (2012). Progranulin: a growth factor, a novel TNFR ligand and a drug target. Pharmacol. Ther..

[4] Zhou M (2015). Progranulin protects against renal ischemia/reperfusion injury in mice. Kidney Int..

[5] Matsubara T (2012). PGRN is a key adipokine mediating high fat diet-induced insulin resistance and obesity through IL-6 in adipose tissue. Cell Metab..

[6] Nicoletto BB, Krolikowski TC, Crispim D, Canani LH (2016). Serum and urinary progranulin in diabetic kidney disease. PLoS ONE.

[7] Nicoletto BB, Canani LH (2015). The role of progranulin in diabetes and kidney disease. Diabetol. Metab. Syndr..

[8] Kamei N (2018). Association between circulating tumor necrosis factor-related biomarkers and estimated glomerular filtration rate in type 2 diabetes. Sci. Rep..

[9] Gohda T, Tomino Y (2013). Novel biomarkers for the progression of diabetic nephropathy: soluble TNF receptors. Curr. Diab. Rep..

[10] Speeckaert MM, Speeckaert R, Laute M, Vanholder R, Delanghe JR (2012). Tumor necrosis factor receptors: biology and therapeutic potential in kidney diseases. Am. J. Nephrol..

[11] Gohda T (2018). Clinical predictive biomarkers for normoalbuminuric diabetic kidney disease. Diabetes Res. Clin. Pract..

[12] Niewczas MA (2012). Circulating TNF receptors 1 and 2 predict ESRD in type 2 diabetes. J. Am. Soc. Nephrol..

[13] Gohda T (2012). Circulating TNF receptors 1 and 2 predict stage 3 CKD in type 1 diabetes. J. Am. Soc. Nephrol..

[14] Sonoda Y (2015). Circulating TNF receptors 1 and 2 are associated with the severity of renal interstitial fibrosis in IgA nephropathy. PLoS ONE.

[15] Murakoshi M (2017). Effect of tonsillectomy with steroid pulse therapy on circulating tumor necrosis factor receptors 1 and 2 in IgA nephropathy. Clin. Exp. Nephrol..

[16] Gohda T (2017). Circulating TNF receptors 1 and 2 Predict mortality in patients with end-stage renal disease undergoing dialysis. Sci. Rep..

[17] Tang W (2011). The growth factor progranulin binds to TNF receptors and is therapeutic against inflammatory arthritis in mice. Science.

[18] Konopka J, Richbourgh B, Liu C (2014). The role of PGRN in musculoskeletal development and disease. Front. Biosci. (Landmark Ed).

[19] Ernandez T, Mayadas TN (2009). Immunoregulatory role of TNFalpha in inflammatory kidney diseases. Kidney Int..

[20] Omote K (2014). Role of the TNF pathway in the progression of diabetic nephropathy in KK-A(y) mice. Am. J. Physiol. Renal Physiol..

[21] Christensen EI, Birn H (2002). Megalin and cubilin: multifunctional endocytic receptors. Nat. Rev. Mol. Cell Biol..

[22] Li H (2014). Circulating PGRN is significantly associated with systemic insulin sensitivity and autophagic activity in metabolic syndrome. Endocrinology.

[23] Richter J (2013). Serum levels of the adipokine progranulin depend on renal function. Diabetes Care.

[24] Kayasuga Y (2007). Alteration of behavioural phenotype in mice by targeted disruption of the progranulin gene. Behav. Brain Res..

[25] Li YS, Song MF, Kasai H, Kawai K (2013). 8-hydroxyguanine in urine and serum as an oxidative stress marker: effects of diabetes and aging. J. Uoeh.

[26] Ward ME (2017). Individuals with progranulin haploinsufficiency exhibit features of neuronal ceroid lipofuscinosis. Sci. Transl. Med..

[27] Wils H (2012). Cellular ageing, increased mortality and FTLD-TDP-associated neuropathology in progranulin knockout mice. J. Pathol..

[28] Kovsan J (2011). Altered autophagy in human adipose tissues in obesity. J. Clin. Endocrinol. Metab..

[29] Yang L, Li P, Fu S, Calay ES, Hotamisligil GS (2010). Defective hepatic autophagy in obesity promotes ER stress and causes insulin resistance. Cell Metab..

[30] Yoo W (2019). Progranulin attenuates liver fibrosis by downregulating the inflammatory response. Cell Death Dis..

[31] Liu J (2015). PGRN induces impaired insulin sensitivity and defective autophagy in hepatic insulin resistance. Mol. Endocrinol..

[32] Kuwahara S (2016). Megalin-mediated tubuloglomerular alterations in high-fat diet-induced kidney disease. J. Am. Soc. Nephrol..

[33] Saito A, Sato H, Iino N, Takeda T (2010). Molecular mechanisms of receptor-mediated endocytosis in the renal proximal tubular epithelium. J. Biomed. Biotechnol..

[34] Weinberg JM (2006). Lipotoxicity. Kidney Int..

[35] Takeyama A (2011). Megalin is downregulated via LPS-TNF-alpha-ERK1/2 signaling pathway in proximal tubule cells. Biochem. Biophys. Res. Commun..

[36] Hu F (2010). Sortilin-mediated endocytosis determines levels of the frontotemporal dementia protein, progranulin. Neuron.

[37] Christensen EI (2007). Distribution of alpha-galactosidase A in normal human kidney and renal accumulation and distribution of recombinant alpha-galactosidase A in Fabry mice. J. Am. Soc. Nephrol..

[38] Prabakaran T (2011). Receptor-mediated endocytosis of alpha-galactosidase A in human podocytes in Fabry disease. PLoS ONE.

[39] Kao AW, McKay A, Singh PP, Brunet A, Huang EJ (2017). Progranulin, lysosomal regulation and neurodegenerative disease. Nat. Rev. Neurosci..

[40] Jian J (2018). Chitinase-3-like protein 1: a progranulin downstream molecule and potential biomarker for Gaucher disease. EBioMedicine.

[41] Chen Y (2018). Progranulin associates with hexosaminidase A and ameliorates GM2 ganglioside accumulation and lysosomal storage in Tay–Sachs disease. J. Mol. Med. (Berl.).

[42] Koussounadis A, Langdon SP, Um IH, Harrison DJ, Smith VA (2015). Relationship between differentially expressed mRNA and mRNA-protein correlations in a xenograft model system. Sci. Rep..

[43] Shebl FM (2010). Comparison of mRNA and protein measures of cytokines following vaccination with human papillomavirus-16 L1 virus-like particles. Cancer Epidemiol. Biomarkers Prev..

[44] Murakoshi M (2011). Mindin: a novel marker for podocyte injury in diabetic nephropathy. Nephrol. Dial. Transplant..

[45] Schmittgen TD, Livak KJ (2008). Analyzing real-time PCR data by the comparative C(T) method. Nat. Protoc..

[46] Zhang WX (2016). *S*election of suitable reference genes for quantitative real-time PCR normalization in three types of rat adipose tissue. Int. J. Mol. Sci..

[47] Cabiati M (2012). Tissue-specific selection of stable reference genes for real-time PCR normalization in an obese rat model. J. Mol. Endocrinol..

[48] Ishizaka M (2015). Podocyte-specific deletion of Rac1 leads to aggravation of renal injury in STZ-induced diabetic mice. Biochem. Biophys. Res. Commun..

[49] Takeda N (2013). Altered unfolded protein response is implicated in the age-related exacerbation of proteinuria-induced proximal tubular cell damage. Am. J. Pathol..

[50] Takaya K (2003). Involvement of ERK pathway in albumin-induced MCP-1 expression in mouse proximal tubular cells. Am. J. Physiol. Renal Physiol..

[51] Kuwagata S (2016). MicroRNA148b-3p inhibits mTORC1-dependent apoptosis in diabetes by repressing TNFR2 in proximal tubular cells. Kidney Int..

