# Supplementary material for: Differential organ-specific inflammatory response to progranulin in high-fat diet-fed mice
Source: Sci Rep. 2021 Jan 13;11:1194. doi: 10.1038/s41598-020-80940-8 (PMC7806827; doi:10.1038/s41598-020-80940-8)
Supplement: Supplementary file 1 — Supplementary Figures. [file 41598_2020_80940_MOESM1_ESM.docx]

Supplementary information

Differential organ-specific inflammatory response to progranulin in high-fat diet-fed mice

Maki Murakoshi, Tomohito Gohda*, Eri Adachi, Saki Ichikawa, Shinji Hagiwara, and Yusuke Suzuki

Supplementary Figure 1. Mean food intake at 8, 12, 16, 20 weeks of age in each group. There is no difference in food intake between the WT-HFD and KO-HFD groups.

Supplementary Figure 2. Immunofluorescence staining for PGRN in PGRN-KO mice.

PGRN is not expressed in the PGRN-KO mice.

Supplementary Figure 3. Oil Red O staining. (A) Kidney sample from an HFD-fed WT mouse. The vacuoles in proximal tubules (closed arrowheads) did not stain with Oil Red O (400×, scale bar: 20 µm). (B) As a reference, liver sample of an HFD-fed WT mouse showing fatty liver changes (200×, scale bar: 50 µm).

Supplementary Figure 4. mRNA expression levels of *Ccl2* and *Tnfrsf1b* in cultured mouse proximal tubule cells stimulated with TNFα. *Ccl2* and *Tnfrsf1b* mRNA expression levels are significantly increased by TNFα stimulation. Student’s unpaired *t* test. *p < 0.05 vs no TNFα stimulation.

Supplementary Figure 5. Knockdown efficiency of siRNA against PGRN. Transfection by PGRN siRNAs reduced the *Grn* mRNA expression levels by approximately 70% compared to the cells transfected with negative control siRNA.
