# Supplementary material for: Differential organ-specific inflammatory response to progranulin in high-fat diet-fed mice
Source: Sci Rep. 2021 Jan 13;11:1194. doi: 10.1038/s41598-020-80940-8 (PMC7806827; doi:10.1038/s41598-020-80940-8)

Supplementary information

Differential organ-specific inflammatory response to progranulin in high-fat diet-fed mice

Maki Murakoshi, Tomohito Gohda*, Eri Adachi, Saki Ichikawa, Shinji Hagiwara, and Yusuke Suzuki

Figure S1

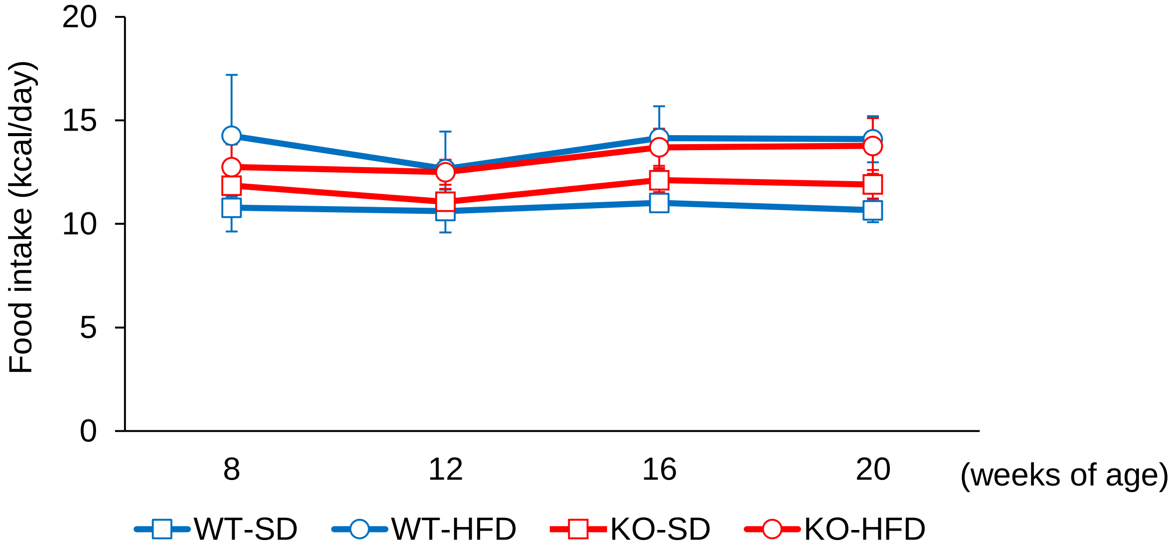

20
15
10
5
0
Food intake (kcal/day)
8
12
16
20
(weeks of age)
WT-SD
WT-HFD
KO-SD
KO-HFD

Figure S2

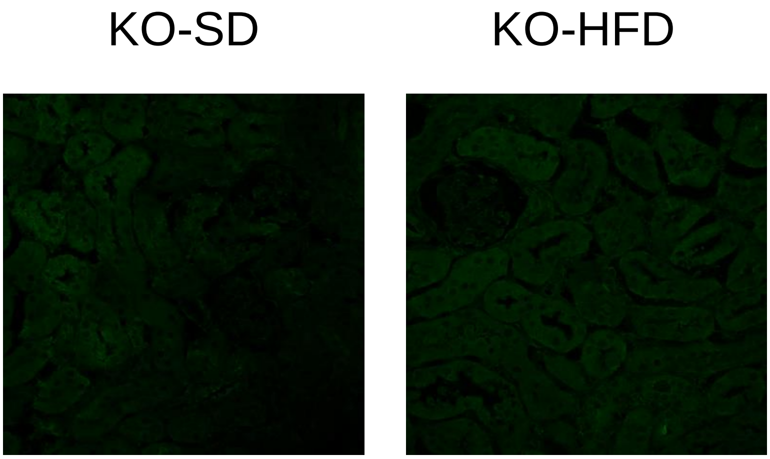

Figure S3

A) Kidney

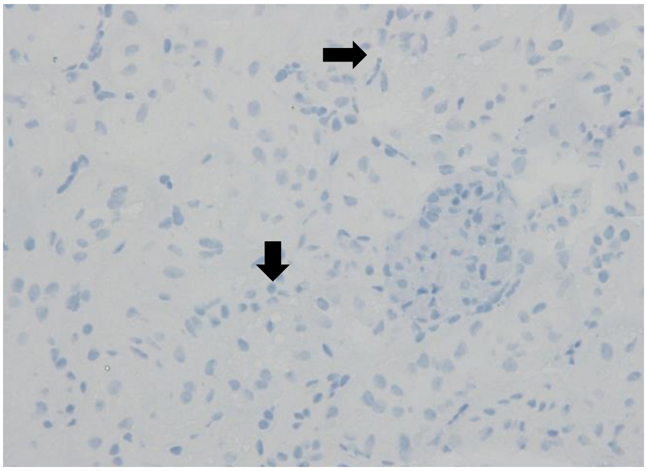

— 20 µm

B) Liver

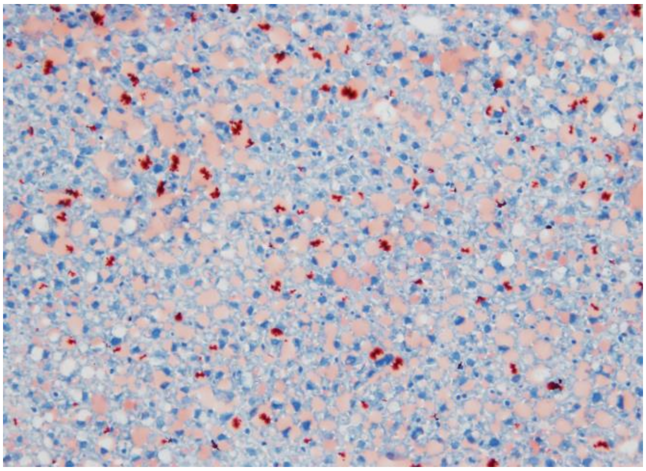

— 50 µm

Figure S4

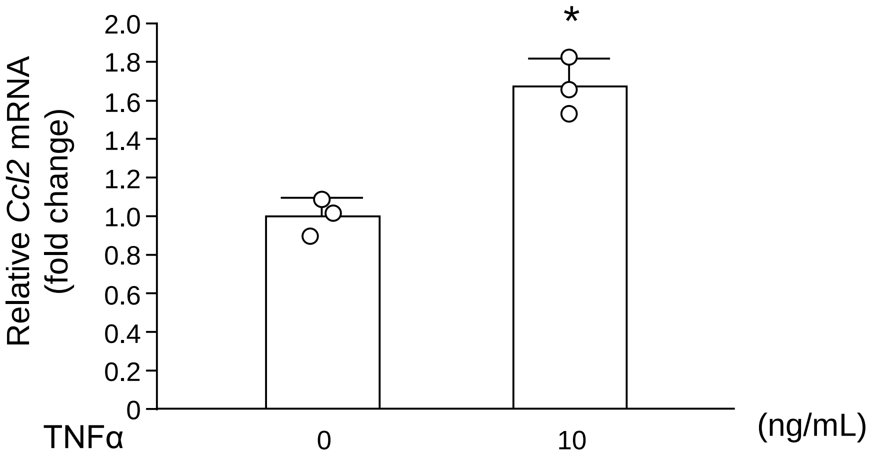

Relative *Ccl2* mRNA (fold change)
2.0
1.8
1.6
1.4
1.2
1.0
0.8
0.6
0.4
0.2
0
*
TNFα
0
10
(ng/mL)

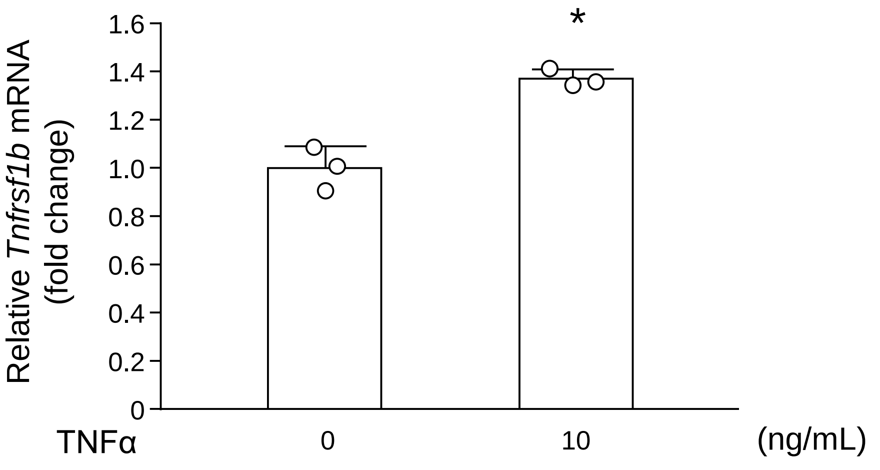

Relative *Tnfrsf1b* mRNA (fold change)
1.6
1.4
1.2
1.0
0.8
0.6
0.4
0.2
0
*
TNFα
0
10
(ng/mL)

Figure S5

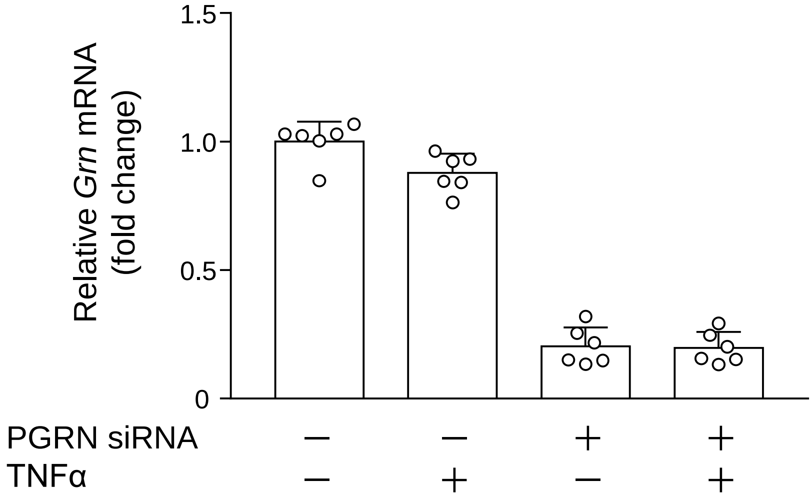

Supplement: Supplementary file 2 — Supplementary Legends. [file 41598_2020_80940_MOESM2_ESM.pdf]
